# Incomplete Augmented Lagrangian Preconditioner for Steady Incompressible Navier-Stokes Equations

**DOI:** 10.1155/2013/486323

**Published:** 2013-10-22

**Authors:** Ning-Bo Tan, Ting-Zhu Huang, Ze-Jun Hu

**Affiliations:** ^1^School of Mathematical Sciences, University of Electronic Science and Technology of China, Chengdu, Sichuan 611731, China; ^2^Department of Information and Computational Science, Chengdu Technological University, Chengdu, Sichuan 611730, China

## Abstract

An incomplete augmented Lagrangian preconditioner, for the steady incompressible Navier-Stokes equations discretized by stable finite elements, is proposed. The eigenvalues of the preconditioned matrix are analyzed. Numerical experiments show that the incomplete augmented Lagrangian-based preconditioner proposed is very robust and performs quite well by the Picard linearization or the Newton linearization over a wide range of values of the viscosity on both uniform and stretched grids.

## 1. Introduction

We consider the numerical solution for large systems of linear equations that arise from the finite element discretization of the incompressible steady-state Navier-Stokes equations governing the flow of viscous Newtonian fluids. The primitive variables formulation of the steady Navier-Stokes equations are

(1) $$\begin{array}{c} -\nu \Delta u+\left(u\cdot \Delta \right)u+\nabla p=f\;\mathrm{on}\;\Omega \times \left[0,T\right],\\ \mathrm{div}\;u\;\mathrm{on}\;\Omega \times \left[0,T\right],\\ u=g\;\mathrm{on}\;\partial \Omega \times \left[0,T\right],\\ u\left(x,0\right)=u_{0}\left(x\right)\;\mathrm{on}\;\Omega , \end{array}$$

where *Ω* ⊂ *R* ^2^ is an open bounded domain with sufficiently smooth boundary ∂*Ω*, [0, *T*] is the time interval of interest, **u**(**x**, *t*) and *p*(**x**, *t*) are the unknown velocity and pressure fields, *ν* is the kinematic viscosity, Δ is the vector Laplacian, ∇ is the gradient, div the divergence, and **f**, **g**, and **u**_0_ are given functions.

We refer to [1] for an introduction to the numerical solution of the Navier-Stokes equations. Implicit time discretization and linearization of the Navier-Stokes equations by the Picard fixed iteration result in a sequence of steady Oseen problems. Spatial discretization of the steady Oseen problems using LBB-stable finite elements (see [1, 2]) is reduced to a series of large sparse linear systems of equations with the following saddle point matrix structure:

(2) $$\begin{array}{c} \tilde{A}x=b, \end{array}$$

with

(3) $$\begin{array}{c} \tilde{A}=\left(\begin{array}{cc} A & B^{T}\\ B & 0 \end{array}\right),\;\;x=\left(\begin{array}{c} u\\ p \end{array}\right),\;\;b=\left(\begin{array}{c} f\\ g \end{array}\right), \end{array}$$

where **u** and *p* represent the discrete velocity and pressure, respectively. This matrix is positive definite, in the sense that **A** + **A** ^*T*^ is symmetric positive definite.

In the past few years, a considerable amount of work has been spent in developing efficient preconditioners for incompressible flow problems; see [1, 3] for a comprehensive survey. Most of the classical and recent preconditioners are based on approximate block factorization. This class includes a variety of block diagonal and block triangular preconditioners. The crucial ingredient in all these methods is an approximation to the Schur complement. This class includes the pressure convection diffusion (PCD) preconditioner, the least squares commutator (LSC) preconditioner, and their variants [4–6]. Another approach is based on the Hermitian or skew Hermitian splitting (HSS) (see [7–10]), and dimensional splitting (DS) of the problem along the components of the velocity field and its relaxed version are introduced in [11, 12]. More recently, preconditioners for incompressible flow problem based on the augmented Lagrangian (AL) reformulation of (2) have been introduced and analyzed in [13–17].

The remainder of the paper is organized as follows. In Section 2, we present a new preconditioner based on the incomplete augmented Lagrangian formulation and a study of the spectrum of the preconditioned system. In Section 3, we show the results of a series of numerical experiments indicating that the incomplete augmented Lagrangian-based preconditioner has been implemented efficiently for the steady incompressible Navier-Stokes equations.

## 2. Incomplete AL-Based Preconditioner for Stable Finite Elements

In this section, we introduce the incomplete AL-base preconditioner for the steady Oseen problem discretized by stable finite element pairs, such as Q2-Q1 or Q2-P1. Here, we consider 2D problems as follows:

(4) $$\begin{array}{c} \left(\begin{array}{ccc} A_{1} & 0 & B_{1}^{T}\\ 0 & A_{2} & B_{2}^{T}\\ B_{1} & B_{2} & 0 \end{array}\right)\left(\begin{array}{c} u\\ v\\ p \end{array}\right)=\left(\begin{array}{c} f_{1}\\ f_{2}\\ g \end{array}\right), \end{array}$$

where *A*_1_ ∈ *R* ^*n*_1_×*n*_1_^, *A*_2_ ∈ *R* ^*n*_2_×*n*_2_^, *B*_1_ ∈ *R* ^*m*×*n*_1_^, and *B*_2_ ∈ *R* ^*m*×*n*_2_^. Thus, *A* ∈ *R* ^*n*×*n*^ with *n* = *n*_1_ + *n*_2_. It is often possible to use augmented Lagrangian techniques to replace the original saddle point system with an equivalent one having the same solution; the original system given in (4) is replaced by incomplete augmented Lagrangian form

(5) $$\begin{array}{c} \left(\begin{array}{ccc} A_{1} & 0 & B_{1}^{T}\\ \gamma B_{2}^{T}W^{-1}B_{1} & {\hat{A}}_{2} & B_{2}^{T}\\ B_{1} & B_{2} & 0 \end{array}\right)\left(\begin{array}{c} u\\ v\\ p \end{array}\right)=\left(\begin{array}{c} f_{1}\\ {\hat{f}}_{2}\\ g \end{array}\right)\;\text{or}\;\hat{A}x=\hat{b}, \end{array}$$

where ${\hat{A}}_{2}=A_{2}+\gamma B_{2}^{T}W^{-1}B_{2}$, ${\hat{f}}_{2}=f_{2}+\gamma B_{2}^{T}W^{-1}g$, *W* is SPD, and *γ* > 0. For form (5), the incomplete augmented Lagrangian (or IAL for short) preconditioner is defined as follows:

(6) $$\begin{array}{c} \left(\begin{array}{ccc} A_{1} & 0 & B_{1}^{T}\\ \gamma B_{2}^{T}W^{-1}B_{1} & {\hat{A}}_{2} & B_{2}^{T}\\ -\gamma \hat{S}W^{-1}B_{1} & B_{2} & \hat{S} \end{array}\right)\left(\begin{array}{c} u\\ v\\ p \end{array}\right)=\left(\begin{array}{c} f_{1}\\ {\hat{f}}_{2}\\ g \end{array}\right), \end{array}$$

where $\hat{S}$ is an approximation to the Schur complement $S=-B{\hat{A}}_{\gamma }B^{T}$, ${\hat{A}}_{1}=A_{1}+\gamma B_{1}^{T}W^{-1}B_{1}$, *A*_21_ = *γB*_2_^*T*^ *W* ^−1^ *B*_1_, and ${\hat{A}}_{\gamma }=\left(\begin{array}{cc} {\hat{A}}_{1} & 0\\ 2A_{21} & {\hat{A}}_{2} \end{array}\right)$.  $\hat{S}$ is implicitly defined through its inverse

(7) $$\begin{array}{c} {\hat{S}}^{-1}=-\gamma {\hat{M}}_{p}^{-1}, \end{array}$$

where ${\hat{M}}_{P}$ is the diagonal of the pressure mass matrix *M*_*p*_.

It is important to note that the preconditioner *M* can be written in factorized form as

(8) $$\begin{array}{c} M=\left(\begin{array}{ccc} {\hat{A}}_{1} & 0 & B_{1}^{T}\\ 2A_{21} & {\hat{A}}_{2} & B_{2}^{T}\\ 0 & 0 & \hat{S} \end{array}\right)\left(\begin{array}{ccc} I & 0 & 0\\ 0 & I & 0\\ -\gamma W^{-1}B_{1} & 0 & I \end{array}\right). \end{array}$$

It follows from the identity that

(9) M−1=(I000I0γW−1B10I)(A^1−100−2A^1−1A21A^2−1A^2−1000I) ×(I0B1T0IB2T00−I)(I000I000−S^−1).

**Theorem 1**

Assume that $W={\hat{M}}_{p}$. The preconditioned matrix $T=\hat{A}M^{-1}$ has an eigenvalue at 1 with multiplicity at least *n*_1_ + *n*_2_. The remaining *m* eigenvalues are *λ*_*i*_ of the matrix

(10) $$\begin{array}{c} Z_{\gamma }=\gamma \left({\hat{S}}_{1}+{\hat{S}}_{2}\right)-2\gamma^{2}{\hat{S}}_{2}{\hat{S}}_{1}, \end{array}$$

where ${\hat{S}}_{1}=B_{1}{\hat{A}}_{1}^{-1}B_{1}^{T}{\hat{M}}_{p}^{-1}$ and ${\hat{S}}_{1}=B_{2}{\hat{A}}_{2}^{-1}B_{2}^{T}{\hat{M}}_{p}^{-1}$.

**Proof**

We have

(11) T=A^M−1=(A10B1TγB2TW−1B1A^2B2TB1B20)(I000I0γW−1B10I) ×(A^1−100−2A^1−1A21A^2−1A^2−1000I) ×(I0B1T0IB2T00−I)(I000I000−S^−1)=(I000I0B1A^γ−1B2A^γ−1Zγ),

where

(12) Zγ=−BA^γ−1BTS^−1=γBA^γ−1BTM^p−1=γ(B1B2)(A^1−10−2A^2−1A21A^1−1A^2−1)(B1TB2T)M^p−1=γ(B1A^1−1B1T+B2A^2−1B2T   −2B2A^2−1A21A^1−1B1T)M^p−1=γ(B1A^1−1B1T+B2A^2−1B2T   −2B2A^2−1B2TM^p−1B1A^1−1B1T)M^p−1=γ(S^1+S^2−2γS^2S^1).

Therefore, we can see that the eigenvalues of *T* are given by 1 with multiplicity at least *n*_1_ + *n*_2_, and the remaining *m* eigenvalues are *λ*_*i*_ of the matrix *Z*_*γ*_.

**Lemma**

Let $H=\left(\begin{array}{cc} A_{1} & -A_{21}^{T}\\ A_{21} & A_{2} \end{array}\right)\in R^{n\times n}$, *n* = *n*_1_ + *n*_2_, *A*_1_ ∈ *R* ^*n*_1_×*n*_1_^, *A*_2_ ∈ *R* ^*n*_2_×*n*_2_^, and *A*_1_, *A*_2_ be positive definite. Then, *H* is positive definite.

**Lemma**

Let *H* ∈ *R* ^*n*×*n*^ and *B* ∈ *R* ^*m*×*n*^ (*m* ≤ *n*). Let *γ* ∈ *R* and assume that matrices *H*, *H* + *γB* ^*T*^ *W* ^−1^ *B*, *BH* ^−1^ *B* ^*T*^, and *B*(*H* + *γB* ^*T*^ *W* ^−1^ *B*)*B* ^*T*^ are all invertible. Then,

(13) $$\begin{array}{c} {\left[B\left(H+\gamma B^{T}W^{-1}B\right)B^{T}\right]}^{-1}=\left(BH^{-1}B^{T}\right)+\gamma W^{-1}. \end{array}$$

We note that the conditions of Lemma 3 are satisfied if we assume that *B* has full row rank and *H* is positive definite. Hence, the remaining *m* eigenvalues *λ*_*i*_ are solutions of the generalized eigenproblem

(14) $$\begin{array}{c} \gamma B{\hat{A}}_{\gamma }^{-1}B^{T}\phi_{i}=\lambda_{i}{\hat{M}}_{p}\phi_{i}. \end{array}$$

We note that ${\hat{A}}_{\gamma }=H+\gamma B^{T}W^{-1}B$, and Lemma 3 implies

(15) γλi−1ϕi=(BA^γ−1BT)−1M^pϕi=(B(H+γBTW−1BT)−1BT)−1M^pϕi=((BH−1BT)−1+γW−1)M^pϕi=((BH−1BT)−1M^p+γI)ϕi=μi−1ϕi+γϕi.

Hence,

(16) $$\begin{array}{c} \gamma \lambda_{i}^{-1}=\gamma +\mu_{i}^{-1}, \end{array}$$

where *μ*_*i*_ satisfies the generalized eigenproblem

(17) $$\begin{array}{c} BH^{-1}B^{T}\phi_{i}=\mu_{i}^{-1}{\hat{M}}_{p}\phi_{i}. \end{array}$$

Hence, the nonunit eigenvalues of $\hat{A}M^{-1}$ are given by

(18) $$\begin{array}{c} \lambda_{i}=\frac{\gamma }{\gamma +\mu_{i}^{-1}},\;1\leq i\leq m. \end{array}$$

**Theorem 4**

The eigenvalues *λ*_*i*_ of *Z*_*γ*_ are of the form

(19) $$\begin{array}{c} \lambda_{i}=\frac{\gamma \mu_{i}}{1+\gamma \mu_{i}}, \end{array}$$

where the *μ*_*i*_'s satisfy the generalized eigenvalue problem $BH^{-1}B^{T}\phi_{i}=\mu_{i}^{-1}{\hat{M}}_{p}\phi_{i}$.

Since *H* is positive definite, and ${\hat{M}}_{p}$ is SPD (see [1]), we have that the eigenvalues of (17) are enclosed in rectangle contained in the half-plane *ℜ*(*z*) > 0; using this result and the relation (19), we can conclude that the same is true of the eigenvalues of $\hat{A}M^{-1}$. If we denote by *a*_*i*_ and *b*_*i*_ the real and imaginary parts of *μ*_*i*_, respectively, easy manipulations result in the following expressions for the real and the imaginary parts of *λ*_*i*_:

(20) $$\begin{array}{c} ℜ\left(\lambda_{i}\right)=\frac{\gamma \left(a_{i}+\gamma \left(a_{i}^{2}+b_{i}^{2}\right)\right)}{{\left(\gamma a_{i}+1\right)}^{2}+{\left(\gamma b_{i}\right)}^{2}},\\ ℑ\left(\lambda_{i}\right)=\frac{\gamma b_{i}}{{\left(\gamma a_{i}+1\right)}^{2}+{\left(\gamma b_{i}\right)}^{2}}. \end{array}$$

The following result is an immediate consequence of Theorem 4.

**Theorem**

The remaining *m* eigenvalues *λ*_*i*_ are given by (18), where *μ*_*i*_ = *a*_*i*_ + *b*_*i*_ satisfies (17). The following estimates hold.

(21) $$\begin{array}{c} 0<\underset{i}{\min}\;\frac{\gamma a_{i}}{1+\gamma a_{i}}\leq ℜ\left(\lambda_{i}\right)\leq 1,\\ \left|ℑ\left(\lambda_{i}\right)\right|\leq \underset{i}{\max}\;\frac{\gamma |b_{i}|}{{\left(\gamma a_{i}+1\right)}^{2}+{\left(\gamma b_{i}\right)}^{2}}<\frac{1}{2}. \end{array}$$

**Figure 1:**
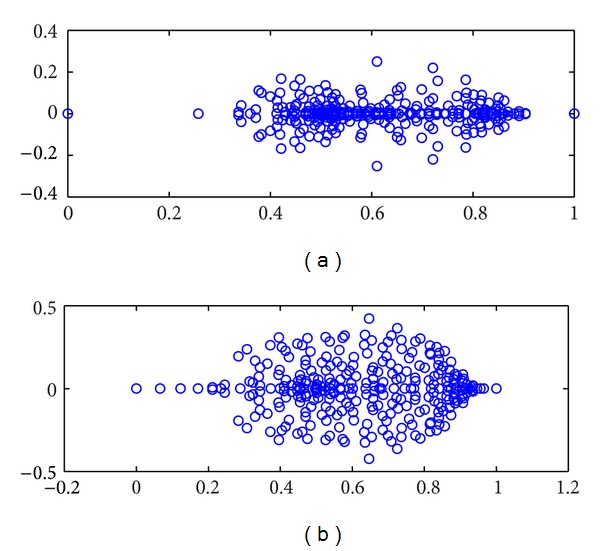
Spectrum of the preconditioned matrix, 32 × 32 grid (Q2-Q1 FEM) (a) *ν* = 0.01, (b) *ν* = 0.001.

Eigenvalue plot of the preconditioned matrices obtained with the incomplete augmented Lagrangian preconditioner is displayed in Figure 1. This plot confirms that, for the incomplete augmented Lagrangian preconditioner, the eigenvalues of the preconditioned matrices are confined to a rectangular region in the half-plane *ℜ*(*z*) ≥ 0; that is, 0 ≤ *ℜ*(*λ*_*i*_) ≤ 1 and |*ℑ*(*λ*_*i*_)|<1/2; note that the appearance of a zero eigenvalue is due to the singularity of the saddle point system (4). In these two examples, corresponding to the viscosities *ν* = 0.01 and *ν* = 0.001, it is clear that the incomplete augmented Lagrangian preconditioner produces a favorable eigenvalue distribution, and the plot shows that the remaining nonzero eigenvalues are well separated from the origin.

## 3. Numerical Experiments

In this section, we will carry out numerical experiments for the linear system coming from the finite element discretization of the two dimensional linearized Stokes and Oseen models of incompressible flow to test the performance of our preconditioner. The test problem is the leaky-lid driven cavity problem generated by the IFISS software package [18]. These experiments were performed in MATLAB on a PC with 2.20 GHz and 2 GB of memory.

Unless otherwise specified, we use right preconditioning with restarted GMRES as the Krylov subspace method, with the maximum subspace dimension set to 30, all these tests are started with an initial guess equal to zero vector. The iteration stops when

(22) $$\begin{array}{c} \frac{{||{\hat{r}}_{k}||}_{2}}{{||\hat{b}||}_{2}}\leq 10^{-6}, \end{array}$$

where **r**_*k*_ is the incomplete augmented Lagrangian system (5) of the residual vector at the *k*th iteration.

We consider the 2D leaky-lid driven cavity problem discretized by the finite elements on uniform grids and stretched grids [1]. The subproblems arising in the application of the incomplete augmented Lagrangian preconditioner are solved by direct methods. We use AMD reordering technique [19, 20] for the degrees of freedom that makes the application of the LU factorization of ${\hat{A}}_{1}$ and ${\hat{A}}_{2}$ relatively fast.

### 3.1. The Leaky Lid Driven Cavity Problem Discretized by Q2-Q1 Finite Elements

The comparison is based on two type test problems. The first type problem is the lid driven cavity problem discretized by Q2-Q1 finite elements with linearization by Picard and Newton on a uniform, respectively. The second type is the same problem but discretized on a stretched grid to investigate the influence of nonuniform elements; the numerical experiments are performed using stretched grids with stretch factors 1.2712 for the 16 × 16 grid, 1.1669 for the 32 × 32 grid, 1.0977 for the 64 × 64 grid, and 1.056 for 128 × 128 grid. The stretching is done in both the horizontal and vertical direction, resulting in rather fine grids near the boundaries.

In Tables 1 and 2, we consider the solution of Picard linearization for the lid driven cavity problem discretized on uniform grids and stretched grids, respectively. For viscosity less than or equal to 0.005, from these results we can see that the performance of the incomplete augmented Lagrangian preconditioner is independent of the mesh size and the viscosity; we also can observe that the uniform grid and stretched grid lead to similar numerical results. Moreover, the optimal *γ* is grid independent and mild dependent viscosity.

Next, we present some results using Newton linearization for the lid driven cavity problem discretized on a uniform grids and stretched grids, respectively. From Tables 3 and 4, it appears that the Newton method gives a similar numerical result on uniform grid and stretched grid, respectively.

### 3.2. The Leaky Lid Driven Cavity Problem Discretized by Q2-P1 Finite Elements

Here, we show results of some tests on problems generated from the discretization using Q2-P1 elements. The preconditioners are tested for a uniform, grid stretched grid, and varying viscosity by Picard or Newton linearization. The numerical results are summarized in Tables 5, 6, 7, and 8. For viscosity not more than 0.005, from these tables we can see again that the convergence rate for the incomplete augmented Lagrangian preconditioner is independent of the mesh size and viscosity; we also can observe that the uniform grid and stretched grid lead to similar numerical results.

### 3.3. Results for the Backward Facing Step Problem

In this subsection, we consider the 2D backward facing step problem using uniform grids. For the step problem, the number of cells in the two directions *x* and *y* is unequal. For this problem, the smallest value of the viscosity used is *ν* = 0.005, since the flow is unsteady for *ν* = 0.001. We show this problem because it is a standard benchmark and because we are interested in seeing the effect of a nonsquare domain. From Tables 9, 10, 11, and 12, we observe iteration counts that are essentially independent mesh size and mildly dependent on the viscosity.

## 4. Conclusions

We have introduced a novel incomplete augmented Lagrangian preconditioner for solving saddle point systems that arise from the finite element discretization of the incompressible steady-state Navier-Stokes equations. We prove that the preconditioned matrix has 1 as an eigenvalue of algebraic multiplicity at least *n* (recall that *n* is the number of velocity degrees of freedom), and the remaining *m* are contained in a box (0,1]×(−1/2, 1/2). Numerical experiments show that the incomplete augmented Lagrangian preconditioner is very robust and performs quite well by Picard linearization or Newton linearization over a wide range of values of the viscosity. The convergence behavior is also quite good for problem posed on stretched grids.

## Figures and Tables

**Table 1:** GMRES iterations with incomplete AL preconditioner for steady Oseen problems (uniform grids, Q2-Q1 FEM, and Picard). The optimal *γ* is in parentheses.

Grid	Viscosity			
	0.1	0.01	0.005	0.001
16 × 16	11 (1.0)	11 (0.08)	15 (0.05)	21 (0.03)
32 × 32	14 (1.0)	13 (0.08)	15 (0.05)	25 (0.03)
64 × 64	14 (1.0)	13 (0.08)	15 (0.05)	29 (0.03)
128 × 128	14 (1.0)	13 (0.08)	15 (0.05)	29 (0.03)

**Table 2:** GMRES iterations with incomplete AL preconditioner for steady Oseen problems (stretched grids, Q2-Q1 FEM, and Picard). The optimal *γ* is in parentheses.

Grid	Viscosity			
	0.1	0.01	0.005	0.001
16 × 16	11 (1.0)	11 (0.08)	12 (0.05)	17 (0.03)
32 × 32	13 (1.0)	12 (0.08)	14 (0.05)	21 (0.03)
64 × 64	14 (1.0)	13 (0.08)	15 (0.05)	26 (0.03)
128 × 128	14 (1.0)	13 (0.08)	16 (0.05)	32 (0.03)

**Table 3:** GMRES iterations with incomplete AL preconditioner for steady Oseen problems (uniform grids, Q2-Q1 FEM, and Newton). The optimal *γ* is in parentheses.

Grid	Viscosity			
	0.1	0.01	0.005	0.001
16 × 16	11 (1.0)	11 (0.08)	12 (0.06)	19 (0.03)
32 × 32	14 (1.0)	12 (0.08)	14 (0.05)	26 (0.03)
64 × 64	14 (1.0)	12 (0.08)	14 (0.05)	29 (0.03)
128 × 128	14 (1.0)	12 (0.08)	14 (0.05)	42 (0.03)

**Table 4:** GMRES iterations with incomplete AL preconditioner for steady Oseen problems (stretched grids, Q2-Q1 FEM, and Newton). The optimal *γ* is in parentheses.

Grid	Viscosity			
	0.1	0.01	0.005	0.001
16 × 16	11 (1.0)	10 (0.08)	11 (0.05)	24 (0.07)
32 × 32	13 (1.0)	11 (0.08)	12 (0.05)	30 (0.05)
64 × 64	14 (1.0)	13 (0.08)	14 (0.05)	45 (0.05)
128 × 128	14 (1.0)	13 (0.08)	14 (0.05)	51 (0.05)

**Table 5:** GMRES iterations with incomplete AL preconditioner for steady Oseen problems (uniform grids, Q2-P1 FEM, and Picard). The optimal *γ* is in parentheses.

Grid	Viscosity			
	0.1	0.01	0.005	0.001
16 × 16	11 (1.0)	12 (0.08)	15 (0.06)	24 (0.03)
32 × 32	11 (1.0)	12 (0.08)	15 (0.06)	26 (0.03)
64 × 64	11 (1.0)	12 (0.08)	15 (0.06)	26 (0.03)
128 × 128	11 (1.0)	11 (0.08)	14 (0.06)	25 (0.03)

**Table 6:** GMRES iterations with incomplete AL preconditioner for steady Oseen problems (stretched grids, Q2-P1 FEM, and Picard). The optimal *γ* is in parentheses.

Grid	Viscosity			
	0.1	0.01	0.005	0.001
16 × 16	11 (1.0)	12 (0.08)	14 (0.06)	26 (0.03)
32 × 32	12 (1.0)	13 (0.08)	16 (0.06)	27 (0.03)
64 × 64	12 (1.0)	12 (0.08)	16 (0.06)	28 (0.03)
128 × 128	12 (1.0)	12 (0.08)	16 (0.06)	28 (0.03)

**Table 7:** GMRES iterations with incomplete AL preconditioner for steady Oseen problems (uniform grids, Q2-P1 FEM, and Newton). The optimal *γ* is in parentheses.

Grid	Viscosity			
	0.1	0.01	0.005	0.001
16 × 16	11 (1.0)	11 (0.08)	12 (0.05)	30 (0.05)
32 × 32	11 (1.0)	10 (0.08)	12 (0.05)	44 (0.05)
64 × 64	11 (1.0)	10 (0.08)	12 (0.05)	46 (0.05)
128 × 128	11 (1.0)	10 (0.08)	11 (0.05)	46 (0.05)

**Table 8:** GMRES iterations with incomplete AL preconditioner for steady Oseen problems (stretched grids, Q2-P1 FEM, and Newton). The optimal *γ* is in parentheses.

Grid	Viscosity			
	0.1	0.01	0.005	0.001
16 × 16	11 (1.0)	11 (0.08)	12 (0.05)	30 (0.05)
32 × 32	12 (1.0)	11 (0.08)	13 (0.05)	46 (0.1)
64 × 64	12 (1.0)	11 (0.08)	13 (0.05)	50 (0.05)
128 × 128	12 (1.0)	10 (0.08)	12 (0.05)	57 (0.05)

**Table 9:** GMRES iterations with incomplete AL preconditioner for the steady Oseen in a uniform backward facing step (Q2-Q1 FEM and Picard).

Grid	Viscosity		
	0.1	0.01	0.005
16 × 48	12 (0.5)	16 (0.2)	20 (0.1)
32 × 96	13 (0.5)	16 (0.08)	19 (0.1)
64 × 182	12 (0.5)	15 (0.08)	21 (0.1)

**Table 10:** GMRES iterations with incomplete AL preconditioner for the steady Oseen in a uniform backward facing step (Q2-Q1 FEM and Newton).

Grid	Viscosity		
	0.1	0.01	0.005
16 × 48	12 (0.5)	15 (0.2)	26 (0.1)
32 × 96	13 (0.5)	16 (0.08)	25 (0.1)
64 × 182	12 (0.5)	15 (0.08)	27 (0.1)

**Table 11:** GMRES iterations with incomplete AL preconditioner for the steady Oseen in a uniform backward facing step (Q2-P1 FEM and Picard).

Grid	Viscosity		
	0.1	0.01	0.005
16 × 48	12 (0.5)	18 (0.2)	25 (0.1)
32 × 96	11 (0.5)	18 (0.2)	23 (0.1)
64 × 182	11 (0.5)	17 (0.2)	22 (0.1)

**Table 12:** GMRES iterations with incomplete AL preconditioner for the steady Oseen in a uniform backward facing step (Q2-P1 FEM and Newton).

Grid	Viscosity		
	0.1	0.01	0.005
16 × 48	12 (0.5)	18 (0.2)	27 (0.1)
32 × 96	11 (0.5)	18 (0.2)	29 (0.1)
64 × 182	11 (0.5)	17 (0.2)	27 (0.1)
